# Hereditary Pancreatic Cancer: Genetic Risk, Surveillance Strategies, and Therapeutic Implications

**DOI:** 10.3390/ijms27146404

**Published:** 2026-07-18

**Authors:** Mariapia Marafioti, Margherita Patruno, Martina Musarra, Nicola Silvestris, Jessica Alejandra Portillo Funes, Fausto Omero, Elena Sapuppo, Vincenzo Cianci, Marco Calabrò, Natasha Irrera, Silvana Briuglia, Mariacarmela Santarpia, Desirèe Speranza

**Affiliations:** 1 School of Specialization in Medical Oncology, Department of Human Pathology “G. Barresi”, University of Messina, 98122 Messina, Italy; marafiotimariapia@gmail.com (M.M.); jessica.a.funes@gmail.com (J.A.P.F.); faustoomero@hotmail.it (F.O.); 2 Division of Oncology “Gaetano Martino” Hospital, University of Messina, 98122 Messina, Italy; martimusa.mm@gmail.com (M.M.); mariacarmela.santarpia@unime.it (M.S.); 3 Center for Study of Heredo-Familial Tumors, Istituto di Ricovero e Cura a Carattere Scientifico (IRCCS) Istituto Tumori “Giovanni Paolo II”, 70124 Bari, Italy; margherita.patruno@gmail.com; 4 Medical Oncology Department, Istituto di Ricovero e Cura a Carattere Scientifico (IRCCS) Istituto Tumori “Giovanni Paolo II”, 70124 Bari, Italy; sapuppo.elena28@gmail.com; 5 Medical Oncology Unit Casa di Cura Villa Salus, 98121 Messina, Italy; 6 Department of Biomedical and Dental Sciences and Morphofunctional Imaging, Section of Legal Medicine, University of Messina, 98125 Messina, Italy; enzocianci.1997@gmail.com; 7 Department of Biomedical and Dental Sciences and Morphofunctional Imaging, University of Messina, 98125 Messina, Italy; mcalabro@unime.it (M.C.); silvana.briuglia@unime.it (S.B.); 8 Department of Clinical and Experimental Medicine, University of Messina, 98125 Messina, Italy; natasha.irrera@unime.it (N.I.); desiree.speranza@gmail.com (D.S.); 9 Department of Human Pathology “G. Barresi”, University of Messina, 98122 Messina, Italy; 10 Department of Chemical, Biological, Pharmaceutical and Environmental Sciences, University of Messina, 98125 Messina, Italy

**Keywords:** pancreatic cancer, hereditary pancreatic cancer, PDAC, germline pathogenic variants, genetic testing, surveillance, targeted treatment

## Abstract

Pancreatic ductal adenocarcinoma (PDAC) remains one of the most lethal malignancies, with a rising incidence and a poor prognosis that largely reflects late-stage diagnosis. Although most cases are sporadic, approximately 5–10% of PDACs occur in the context of inherited cancer susceptibility, including hereditary pancreatic cancer (HPC) syndromes and familial pancreatic cancer (FPC). Germline pathogenic variants in genes involved in DNA damage repair, cell-cycle regulation, and genomic stability—such as *BRCA1*, *BRCA2*, *PALB2*, *ATM*, *CDKN2A*, *STK11*, mismatch repair genes, and *TP53*—contribute to PDAC risk and may influence disease biology. This review provides an overview of the genetic landscape of hereditary and FPC, focusing on established cancer predisposition syndromes and emerging susceptibility genes. Current evidence regarding the prevalence, penetrance, and clinical relevance of germline pathogenic variants (PGVs) is summarized, together with the challenges associated with identifying individuals at increased risk. Contemporary recommendations for germline genetic testing, including the use of multigene panel approaches and limitations in real-world implementation, are also discussed. In addition, surveillance strategies for PDAC in high-risk individuals (HRI) are reviewed, and the available data on the outcomes and limitations of surveillance programs are examined. Finally, the therapeutic implications of inherited alterations, particularly in DNA repair-deficient PDAC, are outlined with reference to genotype-informed systemic treatment approaches.

## 1. Introduction

Pancreatic adenocarcinoma is the third leading cause of cancer death globally with mortality continuing to rise annually. In the United States, an estimated 67,440 new cases of PDAC are projected for 2025. The 5-year survival rate is about 13%, ranging from ~44% in localized disease to ~3% in metastatic stage, highlighting the correlation between higher stage and poorer outcomes [1]. Despite advances in systemic treatments and increased availability of active agents, median overall survival (OS) for metastatic PDAC remains below one year [2,3,4,5]. Only a minority of patients present with resectable tumors at diagnosis, with curative surgery being feasible in approximately 16% of cases [6], highlighting the urgent need for early detection. Nevertheless, population-based screening is currently not feasible, owing both to the relatively low incidence of the disease and to the absence of sufficiently sensitive and specific diagnostic tools [7,8]. These limitations are further compounded by an incomplete understanding of the factors that modulate individual risk for PDAC. Currently, risk factors can be classified as non-modifiable, typically lifestyle and environmental factors, or modifiable, linked to genetic background of individuals [9,10,11,12]. PDAC may arise in sporadic, familial, or hereditary settings. Familial pancreatic cancer (FPC) is generally defined as the occurrence of a PDAC in at least two first-degree relatives in the absence of a known hereditary cancer syndrome. In contrast, hereditary pancreatic cancer (HPC) refers to a PDAC occurring in individuals carrying pathogenic germline variants (PGVs) associated with established cancer predisposition syndromes. Distinguishing between familial aggregation and genetically defined hereditary syndromes is essential for accurate risk assessment, genetic counseling, and the identification of candidates for surveillance programs. Although hereditary and familial forms account for only a minority of PDAC cases, their identification is of major clinical relevance. Traditional clinical and family-history-based criteria have proven insufficient to reliably identify all individuals with an underlying genetic susceptibility. Consequently, since 2019, universal genetic testing has been recommended by the National Comprehensive Cancer Network (NCCN) guidelines [13], yet its implementation in routine clinical practice remains suboptimal [14]. Improving the identification of individuals at increased risk is of critical importance, as it enables enrollment in dedicated surveillance programs, which may facilitate earlier diagnosis and improve clinical outcomes. Although optimal eligibility criteria and surveillance protocols are still under active investigation, targeted screening of high-risk individuals (HRIs), including subjects with hereditary cancer syndromes or significant familial clustering of PDAC, represents a particularly promising approach, given that surgical resection remains the only potentially curative treatment and prognosis for advanced disease continues to be dismal [14,15,16,17,18]. In this context, a deeper understanding of genetic risk factors and hereditary cancer syndromes associated with PDAC is essential to refine risk stratification, optimize screening strategies, and ultimately improve prognosis.

In this review, we first examine the hereditary and familial conditions associated with increased PDAC risk and the underlying susceptibility genes. We then discuss strategies for identifying high-risk individuals, current recommendations for germline genetic testing, surveillance approaches, and the emerging implications of inherited susceptibility for therapeutic decision-making.

## 2. Hereditary and Familial Pancreatic Cancer Risk

Approximately 10% of PDAC cases are attributable to genetic susceptibility. PDACmay occur in individuals with hereditary cancer syndromes caused by PGVs representing ~3% of all cases, denoted as HPC. Another 7% of patients have significant family history but do not carry known hereditary syndromes; these cases are classified as FPC [19]. Several hereditary cancer syndromes and inherited genetic conditions have been associated with an increased risk of PDAC. These syndromes differ in their genetic basis, penetrance, associated cancer spectrum, and magnitude of PDAC risk.

More than 50 hereditary cancer syndromes have been described in humans, a subset of which is associated with PDAC risk [20]. Most HPCs associated with PGVs follow an autosomal dominant inheritance pattern, giving a 50% chance of being passed to offspring. Carcinogenesis usually requires the inactivation of both alleles of a tumor suppressor gene, often along with alterations in additional driver genes. The second allele is commonly inactivated through somatic mutations, loss of heterozygosity, or epigenetic silencing via promoter hypermethylation. However, in some PGVs carriers, PDAC can arise independently of the inherited mutation [21]. Yurgelun et al. [21] reported evidence of a tumor second hit in only 44.4% of pathogenic germline variant carriers. Interpretation of this finding is limited by the study design, which did not include comprehensive assessment of alternative mechanisms of biallelic inactivation, such as promoter methylation or copy-number alterations [21]. These findings emphasize the need for further research to understand the malignant progression associated with distinct germline mutations. Clinically relevant hereditary syndromes associated with PDAC include Peutz–Jeghers syndrome (STK11; ~40-fold risk), hereditary pancreatitis (PRSS1; up to 60-fold risk), CDKN2A-associated FAMMM syndrome (13–22-fold risk), hereditary breast and ovarian cancer syndrome due to BRCA1/2 or PALB2 variants (2–10-fold risk), Lynch syndrome (MMR genes; ~8.6-fold risk), and familial adenomatous polyposis (APC; ~4.5-fold risk) [22,23,24,25].

**Figure 1:**
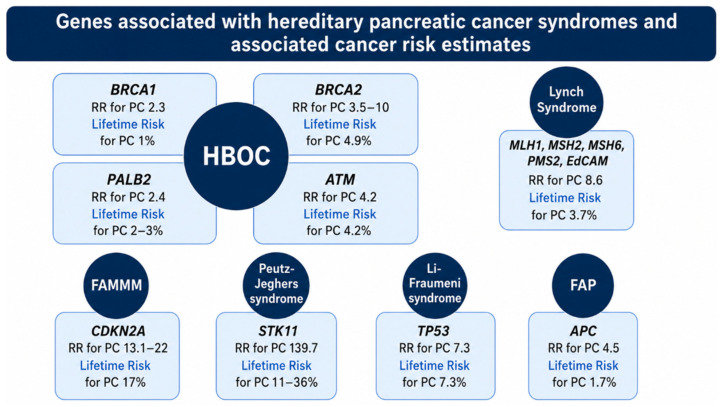
Genes associated with hereditary pancreatic cancer (HPC) and risk estimates. This figure illustrates the major hereditary cancer syndromes associated with an increased risk of pancreatic cancer, along with their corresponding causative genes. For each syndrome, the reported relative risk (RR) and cumulative lifetime risk estimates for pancreatic cancer are shown, highlighting the heterogeneity in genetic predisposition and cancer risk across different inherited conditions. PC: pancreatic cancer; RR: relative risk; HBOC: hereditary breast and ovarian cancer syndrome; FAMMM: familial atypical multiple mole melanoma syndrome; FAP: familial adenomatous polyposis [26].

The identification of these syndromes has important implications for genetic counseling, individualized risk stratification, determination of surveillance eligibility, and emerging targeted therapeutic strategies. The following sections provide a detailed overview of the principal hereditary conditions associated with PDAC risk (Figure 1).

### 2.1. Hereditary Pancreatitis

Recurrent pancreatic injury, as observed in HP, is a well-established risk factor for pancreatic carcinogenesis. Hereditary pancreatitis is defined as pancreatitis occurring in two or more affected individuals across at least two generations within a family, or pancreatitis associated with PGVs in the cationic trypsinogen gene *PRSS1* (protease serine 1). The disorder follows an autosomal dominant inheritance pattern. In addition to *PRSS1*, variants in the *serine protease inhibitor Kazal type 1* (*SPINK1*) and the *cystic fibrosis transmembrane conductance regulator* (*CFTR*) genes have been identified as causative or disease-modifying factors [27]. Pathogenic *PRSS1* variants are present in up to 80% of individuals with HP and promote premature trypsinogen autoactivation within the pancreas, resulting in recurrent acute episodes and progression to chronic pancreatitis. Clinical manifestations typically begin early in life, with most patients developing symptoms before the age of 20 years [28]. Although HP is rare, with a prevalence of less than 1 per 100,000 individuals [29], affected patients face a markedly elevated risk of PDAC. While HP accounts for only a small fraction of all PDAC cases, pooled analyses have demonstrated an exceptionally high relative risk, estimated at 69 compared with the general population [30]. Earlier estimates of the lifetime risk of PDAC among individuals with HP ranged from 18.8% to 53.5% [31,32,33]. However, more recent data from the United States suggest a substantially lower cumulative risk. In a cohort analyzed by Shelton et al., the estimated lifetime risk of PDAC was 7.2% [34]. Consistent findings emerged from population-based analyses, including data derived from the SEER (Surveillance, Epidemiology, and End Results) registry, which evaluated 112 affected families in Europe. These studies reported a lifetime PDAC risk ranging from 7.2% to 18.8%, with relative risk estimates between 59 and 67 [31,32,34]. Notably, both studies demonstrated a sharp increase in PDAC risk after the age of 50 years, with particularly elevated risk among smokers and individuals with diabetes mellitus [35]. Data from the EUROPAC study, which included 112 families from 14 countries, identified *PRSS1* PGVs in 81% of affected families. In this cohort, the cumulative risk of PDAC by 70 years of age following disease onset was estimated at 44%, with a standardized incidence ratio of 67 [32]. More recent investigations in the United States and Japan have confirmed an increased PDAC risk in HP, although at lower levels than previously reported. The cumulative risk by age 70 was estimated at 7.2% in the U.S. cohort and 22.8% in the Japanese cohort [34,36]. Several factors have been proposed to explain discrepancies across studies, including differences in patient survival to older ages, referral bias, improved recognition of previously unidentified genetic variants, and changes in environmental exposures. Notably, tobacco smoking has been shown to accelerate the onset of PDAC by approximately two decades, whereas alcohol consumption primarily contributes to pancreatitis development rather than directly increasing cancer risk [37]. As a preventive strategy in selected patients with HP-associated chronic pancreatitis and refractory pain, early total pancreatectomy with islet autotransplantation has been proposed to reduce PDAC risk while preserving endocrine function [38].

### 2.2. Hereditary Breast and Ovarian Cancer

Hereditary breast and ovarian cancer syndrome is caused by germline loss-of-function variants in the tumor suppressor genes *BRCA1* and *BRCA2.* The protein products of these genes interact with key recombination and DNA repair proteins in pathways responsible for maintaining chromosomal integrity, thereby playing a critical role in the regulation of cell division. Loss of *BRCA1* or *BRCA2* function leads to genomic instability and promotes carcinogenesis [39]. A central mediator of this process is RAD51, a protein essential for homologous recombination (HR) repair, which is tightly regulated by *BRCA1* and *BRCA2*. In normal cells, *BRCA1/2* facilitate RAD51 recruitment to sites of DNA damage, ensuring accurate repair. In HBOC-associated tumors, including *BRCA1/2*-mutated pancreatic and breast cancers, dysregulated RAD51 activity contributes to genomic instability. Moreover, RAD51 status influences the tumor’s sensitivity to DNA-damaging agents and Poly (ADP-ribose) polymerase inhibitors (PARPi), representing both a mechanistic link to carcinogenesis and a potential therapeutic vulnerability [40]. Carriers of *BRCA1/2* PGVs have a markedly increased lifetime risk of developing breast and ovarian cancers, with cumulative risks by age 70 estimated at 47–66% for breast cancer and 40–57% for ovarian cancer. In addition, these individuals are at elevated risk for several other malignancies, including PDAC, prostate cancer, male breast cancer and melanoma [41,42,43].

Evidence from familiar PDAC registry studies indicates that *BRCA2* PGVs are present in approximately 5–17% of patients with FPC, whereas *BRCA1* PGVs are considerably less prevalent in this population. Consistently, multiple studies have demonstrated a significant contribution of *BRCA2* to susceptibility to HPC, while failing to identify a substantial role for *BRCA1* PGVs in most FPC cohorts. Overall, germline *BRCA1/2* PGVs are detected in approximately 5–9% of unselected patients with PDAC; among HPC cases, *BRCA2* PGVs represent the most common genetic cause. Accordingly, lifetime risk estimates for PDAC are consistently higher in *BRCA2* carriers (3.0–7.0%) compared with *BRCA1* carriers (2.2–3.0%) [44,45,46,47,48,49]. A PDAC cluster region has been identified within the central portion of *BRCA2* (c.3249–7471), overlapping with the ovarian cancer cluster region, further supporting a shared molecular basis for cancer predisposition in HBOC families [50]. Epidemiological studies have reported a statistically significant 2.4-fold increase in PDAC incidence among *BRCA* carriers. In families harboring *BRCA2* PGVs in which at least one individual developed early-onset PDAC (<50 years), the risk of PDAC in subsequent generations was estimated to be nearly tenfold higher than that of the general population [51]. Clinical observations suggest that *BRCA*-mutated PDAC tends to occur later in life than *BRCA*-mutated breast cancer, with reported median ages of onset of 53.5 and 45.0 years, respectively (*p* = 0.050). These findings support the rationale for early genetic testing at the time of breast cancer diagnosis and long-term surveillance of *BRCA1/2* carriers to enable detection of PDAC at a potentially resectable stage [52]. The prevalence of newly identified *BRCA1/2* PGVs also varies by ethnicity, with higher rates reported among African-American patients (10.7%) compared with Caucasian (6.1%), Asian (5.0%), and other populations (1.6%). In the general population, deleterious *BRCA* variants occur in approximately 0.2–0.3%, with substantially higher prevalence observed in specific subgroups, such as individuals of Ashkenazi Jewish ancestry (≈2%) [53]. The prognostic impact of germline *BRCA* PGVs on PDAC survival remains under investigation. However, encouraging results have been reported for targeted therapeutic approaches, particularly PARPi, which have demonstrated efficacy in *BRCA*-mutated breast and ovarian cancers and are currently being evaluated in germline-mutated PDAC [54].

#### PALB2

In recent years, the *PALB2* gene has attracted increasing scientific interest owing to its biological function. *PALB2* encodes a protein that interacts with and stabilizes *BRCA2* within the nucleus and plays an essential role in the repair of DNA double-strand breaks. It is currently regarded as the third-most-important gene associated with hereditary breast cancer, following *BRCA1* and *BRCA2*. PGVs in *PALB2* are associated with a moderate increase in breast cancer risk (odds ratio: 3.83) [55], as well as with elevated risks of ovarian cancer (relative risk (RR): 2.91) and PDAC (RR: 2.37). The cumulative risk of PDAC by 80 years of age has been estimated to reach approximately 4% [56]. Additionally, monoallelic inactivation of *PALB2* confers an increased susceptibility to a range of malignancies, including PDAC [57]. Germline *PALB2* pathogenic variants have been identified in approximately 1–2% of familial breast cancer cases and in 2–3% of HPC cases. In a focused analysis of 94 breast cancer patients without *BRCA1* or *BRCA2* PGVs but with a personal or family history of PDAC, the prevalence of *PALB2* PGVs was 2.1% [58]. Although the lifetime risk of PDAC associated with *PALB2* PGVs is estimated to remain below the 5% threshold commonly used to define HRI, this risk may be higher among carriers with a first-degree relative affected by PDAC. Overall, *PALB2* PGVs appear to be relatively uncommon in PDAC. In a study by Tischkowitz et al., pathogenic *PALB2* variants were identified in fewer than 1% of a cohort of 254 patients with PDAC [59]. In contrast, among families with a history of PDAC, the prevalence of *PALB2* PGVs increases to approximately 4% [60].

### 2.3. Familial Atypical Multiple Mole Melanoma

Familial atypical multiple mole melanoma (FAMMM) syndrome is an autosomal dominant genodermatosis characterized by multiple melanocytic nevi, usually more than 50, and a family history of melanoma. It is associated with PGVs in the *CDKN2A* gene and shows reduced penetrance and variable expressivity. Some FAMMM kindreds show an increased risk for the development of PDAC and possibly other malignancies [61]. Individuals from FAMMM families, in addition to a high risk of melanoma, have been estimated to carry a 13- to 22-fold increased risk of developing PDAC compared with the general population [62]. Data from the Melanoma Genetics Consortium (GenoMEL) further demonstrated that the occurrence of PDAC in individuals with familial melanoma is a strong predictor of an underlying pathogenic *cyclin-dependent kinase inhibitor 2A* (*CDKN2A*) variant.

The *CDKN2A* gene encodes two tumor suppressor proteins, p16^INK4a^ and p14^ARF^, which play key roles in cell-cycle regulation. PGVs in *CDKN2A* represent the most frequently identified genetic alteration in hereditary melanoma, also known as FAMMM syndrome [63]. The p16^INK4a^ protein functions as a negative regulator of cell-cycle progression by inhibiting cyclin D/CDK4-mediated transition at the G1/S checkpoint [64]. Carriers of germline *CDKN2A* PGVs exhibit a markedly elevated lifetime risk of cutaneous melanoma, estimated at approximately 70% [65]. Pathogenic variants affecting the p16^INK4a^ protein (CDKN2A/p16) are also strongly associated with an increased risk of PDAC. In a large cohort of Dutch CDKN2A/p16 mutation carriers (the so-called p16-Leiden mutation), Vasen et al. reported a cumulative PDAC incidence of 19% by 70 years of age [66]. For this founder mutation, the relative risk of PDAC has been estimated at 47.8 (95% confidence interval (CI), 28.4–74.7) compared with the general population [67]. Germline *CDKN2A* variants are rare in the general population, with a prevalence below 0.01% [68]. Among unselected PDAC probands, reported mutation frequencies range from 0.3% to 4%, largely depending on the degree of familial aggregation and the presence of additional genetic risk factors in specific geographic regions [69]. In selected patients with early-stage PDAC, total pancreatectomy may represent a reasonable therapeutic option in this high-risk group [70].

### 2.4. Peutz–Jeghers Syndrome

Peutz–Jeghers syndrome is a rare autosomal dominant hereditary disorder characterized by the association of gastrointestinal hamartomatous polyposis, mucocutaneous pigmentation (around the mouth, eyes, and nostrils, in the perianal area, on the buccal mucosa and on the fingers) and cancer predisposition. The estimated prevalence of PJS ranges from 1 in 50,000 to 1 in 200,000 live births [71]. PJS is primarily caused by PGVs in the *serine/threonine kinase 11* (*STK11*) gene, also known as *LKB1*, which encodes a tumor suppressor protein. *STK11* plays a critical role in the regulation of cell polarity, cellular growth and proliferation, and the DNA damage response. Consequently, loss of *STK11* function results in genomic instability and increased susceptibility to tumorigenesis. The majority of individuals fulfilling the clinical diagnostic criteria for PJS harbor PGVs in the *STK11/LKB1* gene [72]. Clinically, PJS confers a strong predisposition to a wide spectrum of malignancies, including cancers of the breast, colorectum, stomach, small intestine, pancreas, and reproductive organs [73]. The cumulative lifetime risk of developing any cancer in individuals with PJS exceeds 90% [71] reflecting the broad oncologic burden associated with this syndrome. PDAC represents the third-most-common malignancy observed in patients with PJS, with reported lifetime risk estimates ranging from 11% to 55% [72]. Individuals with PJS carry an exceptionally high relative risk of PDAC (RR ≈ 132) compared with the general population [74], as well as a markedly elevated absolute lifetime risk estimated between 11% and 36%. Notably, PDAC in PJS is characterized by an earlier age of onset, with a median diagnosis age of approximately 54 years [71,75]. Among all hereditary cancer syndromes, PJS is associated with the highest reported risk of PDAC. Beyond its role in hereditary disease, *STK11* inactivation also contributes to pancreatic carcinogenesis in sporadic settings. Both germline and somatic mutations in *STK11/LKB1* have been identified in pancreatic and biliary tract cancers. Somatic *STK11* mutations occur in approximately 4% of sporadic PDACs and have also been detected in precursor lesions, including intraductal papillary mucinous neoplasms (IPMNs), highlighting the broader role of *STK11* loss in pancreatic tumor development [76,77,78].

### 2.5. Hereditary Non-Polyposis Colorectal Cancer (Lynch Syndrome)

Lynch syndrome (LS) is an autosomal dominant cancer predisposition syndrome caused by PGVs in genes involved in the DNA mismatch repair (MMR) system, including *MutL homolog 1* (*MLH1*), *MutS homolog 2* (*MSH2*), *MutS homolog 6* (*MSH6*), *and post-meiotic segregation increased 2* (*PMS2*) [79]. In addition, large deletions in the *EPCAM* gene may lead to epigenetic silencing of the adjacent *MSH2* gene through transcriptional read-through, resulting in loss of *MSH2* expression and a LS phenotype [80]. Lynch syndrome is characterized by an increased risk for colorectal cancer and cancers of the endometrium, ovary, stomach, small bowel, urinary tract, biliary tract, brain (usually glioblastoma), skin (sebaceous adenomas, sebaceous carcinomas, and keratoacanthomas), pancreas, and prostate. Cancer risks and age of onset vary depending on the associated gene [81].

Multiple studies have demonstrated an elevated risk of PDAC in LS families [82]: the cumulative lifetime risk of PDAC in LS has been estimated at approximately 3.7%, corresponding to an 8.6-fold increased risk compared with the general population [83]. Reported incidence rates of PDAC among LS patients range from 1.3% to 4% with the highest risks observed among *MLH1* and *MSH2* carriers [82,83,84]. While the age at diagnosis of PDAC in LS does not differ significantly from that of sporadic PDAC, it contrasts with the earlier onset observed for several other LS-associated malignancies; the biological basis for this observation remains incompletely understood [82,84]. High level of MSI and mismatch repair protein deficiency detected by immunohistochemistry (dMMR) are hallmark features of Lynch syndrome-associated malignancies. However, these molecular alterations are exceedingly uncommon in PDACs, with reported frequencies below 4%, and in most series under 1%, and have not been observed in FPC cohorts (0/25 cases) [85,86]. Given the rarity of MSI-H and dMMR in PDAC, routine screening using MSI testing or MMR immunohistochemistry on pancreatic tumor tissue is not an efficient strategy for identifying LS. Nevertheless, careful evaluation remains important to avoid missing patients with true MSI-H or dMMR PDACs who may benefit from targeted pharmacotherapeutic approaches.

### 2.6. Familial Adenomatous Polyposis

FAP is a rare autosomal dominant hereditary cancer syndrome caused by germline pathogenic variants in the *APC* gene. Biallelic inactivation of *APC* results in loss of functional APC protein, leading to β-catenin accumulation and constitutive transcriptional activation of the Wnt signaling pathway, a central mechanism in tumorigenesis [87]. Somatic *APC* mutations are also detected in up to 80% of sporadic colorectal adenomas and adenocarcinomas, underscoring the pivotal role of *APC* dysfunction in colorectal carcinogenesis. Approximately 25% of *APC* pathogenic variants arise de novo [88]. In the absence of functional APC tumor suppressor protein, uncontrolled cellular proliferation leads to the development of numerous adenomatous polyps throughout the gastrointestinal tract, predominantly affecting the colorectum and duodenum. Clinically, FAP is characterized by the presence of more than 100 adenomatous polyps, often numbering in the hundreds or thousands. Polyps may also occur in other gastrointestinal sites, including the stomach, duodenum, small intestine, and biliary tract [89]. In addition to gastrointestinal manifestations, FAP is associated with an increased risk of several extraintestinal malignancies, including tumors of the brain, thyroid, and liver [90]. Epidemiological data regarding PDAC in FAP are limited; however, affected individuals appear to have an approximately four- to fivefold increased risk compared with the general population, with a reported relative risk of 4.5 (95% CI, 1.2–11.4) and an estimated absolute lifetime risk of approximately 2% [91]. Despite this increased risk, routine PDAC surveillance for *APC* carriers has not been incorporated into current clinical guidelines. From a preventive standpoint, prophylactic endoscopic polypectomy represents a safe and effective strategy for reducing the risk of duodenal and periampullary carcinoma, anatomical regions closely associated with the pancreatic head [92].

### 2.7. Li–Fraumeni Syndrome (LFS)

Li–Fraumeni syndrome (LFS) is caused by germline pathogenic variants in the *TP53* gene and follows an autosomal dominant pattern of inheritance. The syndrome is rare in the general population, with an estimated prevalence ranging from 0.018% to 0.028% [93]. Loss of normal *TP53* tumor suppressor function predisposes affected individuals to the development of a broad spectrum of malignancies, classically including soft tissue and bone sarcomas, premenopausal breast cancer, leukemia, adrenocortical carcinoma, and primary brain tumors. Carriers of germline *TP53* PVs exhibit a 7.3-fold increased risk of PDAC compared with the general population [94]. By 70 years of age, the cumulative incidence of major LFS-associated malignancies has been estimated at 54% for breast cancer, 15% for soft tissue sarcomas, 6% for brain tumors, and 5% for osteosarcoma [95]. Consistent with these findings, data from the International Agency for Research on Cancer germline *TP53* database indicate that PDAC occurs in approximately 1.2% of individuals with germline *TP53* PVs, with a relatively young median age at diagnosis of 53 years [96].

### 2.8. Ataxia–Telangiectasia (AT)

Ataxia–telangiectasia (AT) is a rare autosomal recessive multisystem disorder caused by biallelic pathogenic variants in the *ATM* gene. Beyond the biallelic syndrome, heterozygous, carriers of a single pathogenic *ATM* variant, do not develop the classical neurological or immunological manifestations of AT, but they nonetheless exhibit a moderate and clinically significant increase in cancer risk [97].

The ATM protein is a serine/threonine kinase that plays a pivotal role in the cellular response to DNA damage, including activation of cell-cycle checkpoints and maintenance of genomic stability. ATM mediates phosphorylation of several key substrates, most notably p53 (encoded by *TP53*) and *BRCA1*, thereby coordinating DNA repair, cell-cycle arrest, and apoptosis following genotoxic stress. Partial loss of ATM function in heterozygous carriers may impair these pathways, promoting genomic instability and tumorigenesis [98,99,100]. Guidelines recommend enhanced breast and prostate surveillance and PDAC monitoring when family history is positive, while prophylactic surgery is generally not indicated, and treatment decisions should not rely solely on *ATM* status. These carriers should undergo personalized surveillance and risk assessment within established clinical frameworks [97]. In a large study including 4607 carriers of pathogenic *ATM* variants and 623,135 control individuals, *ATM* variant carriers showed increased prevalence of multiple malignancies, including invasive ductal breast cancer (33.4%), prostate cancer (32.8%), ovarian cancer (4.4%), colorectal cancer (2.9%), and PDAC (1.4%). The corresponding relative risks were 2.03 (95% CI: 1.89–2.19) for breast cancer, 2.58 (95% CI: 1.93–3.44) for prostate cancer, 1.57 (95% CI: 1.35–1.83) for ovarian cancer, 1.49 (95% CI: 1.24–1.79) for colorectal cancer, and 4.21 (95% CI: 3.24–5.47) for PDAC [101]. Although the precise lifetime risk of PDAC among *ATM* pathogenic variant carriers remains incompletely defined, multiple studies consistently demonstrate a significantly increased risk. Roberts and colleagues first reported the association between germline *ATM* mutations and HPC [102]. Subsequently, a large study of 3030 unselected PDAC cases identified germline *ATM* pathogenic variants in 2.3% of patients [103].

Consistent with these findings, recent clinical data indicate that PDAC risk is more than fourfold higher even among heterozygous *ATM* carriers compared with control subjects (OR 4.21; 95% CI: 3.24–5.47; *p* < 0.0001) [101]. Moreover, *ATM* variants have been identified in approximately 4–9% of pancreatic ductal adenocarcinomas, further supporting the relevance of this gene in pancreatic tumorigenesis [104].

However, due to the wide spectrum of hereditary cancer syndromes linked to PDAC, the identification of individuals eligible for genetic testing remains complex. Family history, combined with a patient’s personal cancer history, represents the cornerstone for identifying inherited cancer predisposition. A three-generation pedigree is considered the gold standard for autosomal inherited disorders and should include tumor types and age at diagnosis. Cancer history in first- and second-degree relatives is particularly relevant. Features suggesting a hereditary component include early age at cancer onset, multiple affected family members, and the presence of multiple primary tumors, especially in organs such as the breast or colon. In addition, ethnicity-related information should be carefully considered, as it may be associated with specific cancer syndromes (Table 1).

## 3. Genetic Testing in Pancreatic Ductal Adenocarcinoma (PDAC)

High-risk individuals (HRIs) are individuals identified as being at increased risk of pancreatic cancer because of a strong family history of PDAC and/or the presence of pathogenic germline variants (PGVs) [9]. Compared with the general population, which has an average lifetime risk of approximately 1.5%, HRIs exhibit a lifetime cumulative risk exceeding 5%, corresponding to at least a fivefold increase in relative risk [108]. Therefore, pancreatic surveillance is recommended for this population according to current international guidelines [9,109]. Genetic testing for hereditary cancer syndromes plays a critical role in identifying individuals at increased risk of PDAC and other associated malignancies who may benefit from tailored surveillance strategies [110].

### 3.1. Guideline Recommendations for Germline Genetic Testing

According to the American Society of Clinical Oncology (ASCO), genetic testing should be offered when a personal or family history is suggestive of a hereditary cancer susceptibility syndrome, when the test can be appropriately interpreted, and when the results have clinical implications for the patient or for at-risk family members [111]. ASCO further states that germline testing may be discussed even in patients with PDAC and no apparent family history [111]. Importantly, up to 41.8% of patients harboring pathogenic germline variants (PGVs) do not meet current eligibility criteria for genetic testing, highlighting limitations of guideline-based approaches. However, genetic testing for inherited PGVs is recommended for all patients with confirmed PDAC, using comprehensive gene panels targeting hereditary cancer syndromes [112,113].

#### 3.1.1. Universal Germline Testing: Rationale and Implementation

Historically, genetic testing was performed selectively following referral to genetic counseling when hereditary cancer was suspected [114]. With the growing support for universal germline testing in PDAC, this traditional approach faces several challenges, including delays in genetic counseling, increased testing demand, and the need for rapid turnaround times. These issues are particularly relevant given the poor prognosis of PDAC and the therapeutic implications of germline findings [115]. In 2019, the NCCN first recommended germline genetic testing for all patients with PDAC [116]. While specific genes were not initially specified, current guidelines endorse an 11-gene panel including *ATM*, *BRCA1*, *BRCA2*, *CDKN2A*, *MLH1*, *MSH2*, *MSH6*, *EPCAM*, *PALB2*, *STK11*, and *TP53* [117].

One key rationale for germline testing is that, although tumor genomic profiling is increasingly incorporated into routine clinical practice in PDAC (particularly in the advanced disease setting), tumor-only sequencing cannot definitively distinguish somatic alterations from germline pathogenic variants (GPVs). Therefore, variants identified as potentially germline through tumor-only profiling require confirmation through dedicated germline testing or matched tumor–normal sequencing [118].

#### 3.1.2. Multigene Panels and Next-Generation Sequencing

Consistent with NCCN guidelines, germline pathogenic variants should be systematically evaluated using comprehensive multigene panels, including *BRCA1*, *BRCA2*, *CDKN2A*, mismatch repair genes associated with LS (*MLH1*, *MSH2*, *MSH6*, *EPCAM*), *ATM*, *PALB2*, *STK11*, and *TP53*. The inclusion of genes associated with HP, specifically *PRSS1* and *SPINK1*, should be determined based on pertinent clinical characteristics [112,113]. Next-generation sequencing-based multigene panel testing has been shown to be more time-efficient and cost-effective than traditional gene-by-gene testing approaches [12]. Although the standard 11-gene panel is widely accepted, several studies have explored the benefit of expanded panels. It has been demonstrated that the inclusion of additional genes such as *CHEK2*, *RAD51C*, and *BRIP1* allows for the identification of pathogenic variants of these genes, which may be a starting point for further studies [119].

#### 3.1.3. Real-World Utilization of Genetic Testing

A retrospective study conducted across three Mayo Clinic sites in the United States demonstrated that rates of genetic counseling, germline testing, and PGV detection remained similar before and after NCCN guideline implementation. Overall, genetic testing discussions occurred in 35–40% of PDAC patients, germline testing was performed in 25–30%, PGVs were detected in 10–20%, and PDAC-associated PGVs were identified in approximately 10% of cases [14].

## 4. Precancerous Lesions

Pancreatic adenocarcinoma is the most common form of PDAC and arises through a multistep carcinogenic process from well-defined precursor lesions, primarily PanINs [120], in approximately 85% of cases [121]. Among cystic pancreatic neoplasms, IPMNs represent a clinically relevant entity, as they may progress from benign lesions to invasive PDAC (IPMN-derived carcinoma) [122], accounting for <10% of all cases [121]. In addition to PanINs and IPMNs, other recognized precursor lesions include mucinous cystic neoplasms, intraductal tubulopapillary neoplasms, and intraductal oncolytic papillary neoplasms (IOPNs) [123] which collectively account for less than 1% of PDACs arising via these pathways [124].

### 4.1. Pancreatic Intraepithelial Neoplasia (PanIN)

PanINs are considered the most important microscopic precursor lesions of PDAC, as most PDACs are thought to arise from these lesions. PanINs are defined as noninvasive epithelial lesions of the pancreatic ducts measuring less than 5 mm, with flat or papillary architecture. Histologically, they consist of columnar-to-cuboidal epithelial cells with variable mucin production and increasing degrees of cytological and architectural atypia.

PanINs follow a well-characterized multistep progression model involving the sequential accumulation of genetic alterations, including activating mutations in *KRAS* and inactivation of *CDKN2A/p16*, *TP53*, and *SMAD4*, as lesions progress from low-grade to high-grade dysplasia [125]. According to current consensus classifications, former PanIN-1A, PanIN-1B, and PanIN-2 lesions are now grouped as low-grade PanIN, whereas the term high-grade PanIN is reserved for lesions with severe cytological and architectural atypia, previously referred to as carcinoma in situ [126].

Given that only a small proportion of low-grade PanIN lesions progress to invasive PDAC, the detection and resection of high-grade PanINs is considered a meaningful surrogate endpoint for surveillance programs.

Although PanINs represent the predominant microscopic precursor lesions of PDAC, accumulating evidence indicates that alternative precursor pathways also exist, particularly in the context of cystic neoplasms such as intraductal papillary mucinous neoplasms (IPMNs), which are discussed in the following section.

### 4.2. Intraductal Papillary Mucinous Neoplasms

IPMNs are grossly visible (>5 mm), noninvasive epithelial neoplasms composed of mucin-producing columnar cells. They can exhibit a spectrum of dysplasia ranging from low-grade to high-grade and may be associated with invasive carcinoma. Morphologically, IPMNs are classified as main-duct (MD-IPMN), branch-duct (BD-IPMN), or mixed-type lesions. MD-IPMNs carry a substantially higher risk of malignant transformation compared with BD-IPMNs (approximately 60% vs. 25%, respectively) [127].

According to international consensus guidelines, features warranting immediate surgical intervention (“high-risk stigmata”) include obstructive jaundice, enhancing mural nodules ≥5 mm, and main pancreatic duct dilation ≥10 mm. “Worrisome features,” such as cyst size ≥3 cm or interval growth, represent relative indications for surgery and require close surveillance [127].

Approximately 15% of PDACs are estimated to arise from IPMNs, underscoring their importance as targets for early detection strategies. Screening studies in high-risk populations have demonstrated a high prevalence of IPMNs, particularly among FPC kindreds, in whom IPMNs are detected in up to 10–18% of individuals [128,129].

Long-term follow-up studies have shown that the cumulative incidence of PDAC after an IPMN diagnosis is approximately 3.3%, 6.6%, and 15% at 5, 10, and 15 years, respectively [130].

IPMNs appear to be more prevalent in individuals with a family history of PDAC or HPC syndromes, suggesting an underlying genetic predisposition [131,132,133,134].

IPMNs are frequently multifocal, further supporting a genetic contribution to their development. However, the association between IPMNs and established PDAC susceptibility genes—including *BRCA1*, *BRCA2*, *CDKN2A*, MMR genes (*MLH1*, *MSH2*, *MSH6*, *PMS2*, *EPCAM*), *ATM*, *PALB2*, *STK11*, and *TP53*—remains incompletely defined. In a cohort of 350 patients with surgically resected IPMNs, germline mutations were identified in 7.3% of patients, with pathogenic variants in established PDAC susceptibility genes present in 2.9% [135]. While the overall prevalence of germline mutations did not differ significantly from that observed in unselected PDAC cohorts, pathogenic *ATM* variants were significantly more frequent among IPMN patients and were associated with a higher likelihood of concomitant PDAC. Consistent with these findings, genomic studies have identified somatic *ATM* alterations in up to 17% of IPMNs and mucinous cystic neoplasms, highlighting the potential role of DNA damage–response pathways in cyst progression and malignant transformation [136].

Molecular studies suggest that PDAC associated with IPMNs may arise through multiple pathways, including stepwise progression from IPMN to carcinoma, clonal divergence, or independent (de novo) tumorigenesis [122].

### 4.3. Other Precursor Lesions and Syndromic Associations

Mucinous cystic neoplasms, intraductal tubulopapillary neoplasms, and IOPNs, although less common, may carry a substantial risk of progression to pancreatic ductal adenocarcinoma and are increasingly recognized through imaging-based surveillance programs. Rare hereditary syndromes, such as McCune–Albright syndrome (associated with *GNAS* PGVs) and Carney complex (associated with *PRKAR1A* PGVs), have also been linked to pancreatic cystic neoplasms and PDAC in limited case series, although their precise contribution to pancreatic carcinogenesis remains incompletely defined.

At the histopathological and molecular level, these precursor lesions follow distinct tumorigenic pathways and display markedly different risks of malignant progression. Mucinous cystic neoplasms exhibit an estimated risk of progression to invasive carcinoma of approximately 10–15%. Intraductal tubulopapillary neoplasms are associated with a high prevalence of invasive carcinoma, identified in up to 70% of cases at diagnosis, whereas intraductal oncocytic papillary neoplasms display intermediate malignant potential, with invasive components observed in approximately 30% of lesions. Collectively, these entities highlight the biological heterogeneity of pancreatic precursor lesions and the variable trajectories leading to invasive PDAC [123,137,138,139,140,141,142].

### 4.4. Implications for Surveillance

Despite the aggressive biology of PDAC, molecular evolutionary studies suggest that the progression from precursor lesions to invasive and metastatic disease occurs over many years, providing a potential window for early detection [143]. This supports the rationale for surveillance programs aimed at identifying high-grade precursor lesions and early-stage PDAC in HRIs.

Although substantial progress has been made in the identification and management of HPC, several challenges remain. Risk estimates associated with individual susceptibility genes vary considerably across studies, reflecting differences in cohort composition, ascertainment strategies, family history burden, and duration of follow-up. Consequently, penetrance estimates should be interpreted with caution, particularly for moderate-risk genes such as ATM and BRCA1, for which available evidence remains less consistent than for high-risk genes such as CDKN2A or STK11. Similarly, surveillance outcomes are heterogeneous across published cohorts. While studies from CAPS and Dutch surveillance programs have demonstrated encouraging rates of early-stage detection and improved survival among high-risk individuals, direct comparisons between surveillance initiatives remain difficult because of differences in eligibility criteria, imaging protocols, surveillance intervals, and outcome definitions. Moreover, many registries have reported limited long-term outcome data, and evidence supporting mortality reduction remains largely derived from observational studies. Another important limitation concerns the incomplete understanding of genotype–phenotype correlations. A substantial proportion of FPC cases do not harbor currently identifiable pathogenic germline variants, suggesting that additional susceptibility genes and modifying factors remain to be discovered. Environmental exposures, particularly tobacco smoking, may further influence individual risk and are not consistently incorporated into current risk prediction models. Future research should therefore focus not only on the identification of novel susceptibility genes but also on the development of integrated risk-stratification models combining genetic, familial, environmental, imaging, and biomarker data. Such approaches may improve patient selection for surveillance and optimize the balance between early detection and overdiagnosis.

The interpretation of surveillance-associated survival benefits also requires caution. Although several surveillance cohorts, including CAPS and Dutch high-risk programs, have reported higher rates of stage I PDAC detection and improved survival compared with historical controls, these findings derive predominantly from observational studies rather than randomized trials. Consequently, the magnitude of the reported benefit may be influenced by several sources of bias. Lead-time bias may artificially prolong measured survival simply because cancer is detected earlier, without necessarily altering the natural history of the disease. Similarly, length-time bias may favor the detection of more indolent lesions with intrinsically better prognoses. Selection bias is also a relevant concern, as individuals enrolled in surveillance programs are often managed at specialized centers, may have greater health awareness, and frequently undergo more intensive clinical follow-up than the general population. Therefore, although current evidence strongly supports the ability of surveillance programs to detect PDAC at earlier and potentially curable stages, the extent to which surveillance independently reduces disease-specific mortality remains an area of ongoing investigation. Long-term prospective studies and continued follow-up of large international cohorts will be essential to better quantify the true clinical benefit of surveillance.

Despite strong recommendations supporting universal or expanded germline testing in PDAC, real-world implementation remains suboptimal and highly heterogeneous across healthcare systems. Reported uptake rates of genetic testing, ranging approximately between 25% and 30% in unselected PDAC populations, highlight a substantial gap between guideline recommendations and clinical practice. Several factors contribute to this implementation gap. First, access to genetic counseling services is uneven, with marked disparities between high-volume tertiary centers and smaller regional hospitals. Limited availability of specialized genetic counselors and oncogenetics clinics often results in delayed or incomplete referral pathways. Second, economic considerations and reimbursement policies vary significantly across healthcare systems, influencing the accessibility of germline testing and associated follow-up services. Third, variability in clinician awareness and familiarity with evolving genetic guidelines further contributes to inconsistent adoption in routine practice. In some settings, germline testing is not systematically integrated into diagnostic or therapeutic workflows, leading to missed opportunities for cascade testing and identification of at-risk relatives. Finally, organizational barriers, including fragmented care pathways and lack of standardized referral protocols, further limit the integration of genetic testing into routine PDAC management. Addressing these barriers will require coordinated efforts to expand access to genetic counseling, standardize testing pathways, improve clinician education, and ensure equitable reimbursement policies. Integration of germline testing into structured registry-based frameworks may represent a key strategy to bridge the gap between evidence and implementation in clinical practice.

## 5. Pancreatic Cancer Surveillance

PDAC surveillance is recommended only in individuals at increased risk, typically defined by genetic predisposition or strong familial aggregation, as there is insufficient evidence to support population-based screening in average-risk individuals [144]. Routine screening in the general population is not recommended due to insufficient sensitivity, specificity, and lack of demonstrated mortality reduction [8]. Therefore, surveillance strategies are reserved for HRIs, generally defined as those with a lifetime risk of PDAC exceeding 5% or a relative risk increase of at least fivefold. Major professional societies and expert consortia, including the American Gastroenterological Association (AGA), the American College of Gastroenterology (ACG), and the International Cancer of the Pancreas Screening Consortium (CAPS), have developed specific recommendations for the surveillance of high-risk individuals [9,109,110].

### 5.1. CAPS Recommendations for PDAC Surveillance

The CAPS Consortium currently represents the most widely adopted international umbrella consortium for PDAC surveillance in high-risk individuals. Successive consensus meetings have provided recommendations regarding eligibility criteria, surveillance modalities, surveillance intervals, and management of detected lesions [145].

The CAPS summit highlighted strong consensus that effective screening should detect margin-negative T1N0M0 PDAC and high-grade precursor lesions. Screening was recommended for selected high-risk groups, including first-degree relatives (FDR) from FPC kindreds, individuals with Peutz–Jeghers syndrome (PJS), and carriers of *p16*, *BRCA2*, or mismatch repairs system (MMR) PGVs with an affected first-degree relative. No agreement was reached on the optimal age to start or stop surveillance, and any recommended surgery should be performed at high-volume centers. Importantly, evidence supporting PDAC surveillance has progressively strengthened. In the multicenter CAPS study, most PDACs detected during surveillance were identified at stage I, and surveillance-detected cases showed substantially improved survival compared with matched controls diagnosed outside surveillance programs, supporting the clinical utility of structured surveillance in high-risk individuals [146].

### 5.2. Registries and High-Risk Cohorts

Over the past decade, multiple national and international registries and high-risk cohorts have significantly advanced the understanding of hereditary and familial PDAC. These initiatives enable longitudinal collection of clinical, genetic, and imaging data, supporting improved risk stratification and the development of surveillance strategies for high-risk individuals [147,148].

In addition, registry-based studies have contributed to the characterization of precursor lesions and their association with pancreatic tumorigenesis in genetically predisposed populations [147].

However, variability in eligibility criteria and surveillance protocols across registries leads to differences in reported outcomes, highlighting the need for harmonization across studies.

#### 5.2.1. Role of Registries in Surveillance

Beyond their role in risk stratification, registry-based cohorts have been essential in translating genetic discoveries into clinical practice. International data indicate that 10–20% of unselected pancreatic cancer patients carry pathogenic germline variants, supporting recommendations for universal germline testing in clinical guidelines [149]. This approach enables the identification of individuals who would not be captured by family history-based criteria and facilitates cascade testing in at-risk relatives, thereby extending preventive strategies to family members.

Multigene panel testing has further expanded the spectrum of clinically relevant germline alterations beyond BRCA1/2, with ATM and BRCA2 among the most frequently implicated genes in selected high-risk populations [150]. Most pathogenic variants involve DNA damage repair pathways, particularly homologous recombination, whereas mismatch repair gene alterations are less frequent, highlighting the heterogeneity of hereditary pancreatic cancer predisposition.

In addition, detailed analyses of precursor lesions have clarified their role in the stepwise evolution of pancreatic cancer in genetically predisposed individuals [151,152].

Importantly, registry data continue to refine genotype–phenotype correlations and penetrance estimates across different genetic backgrounds, supporting more individualized risk assessment and surveillance strategies. Recent analyses from international cohorts have also strengthened the evidence base for integrating germline findings into clinical decision-making frameworks for pancreatic cancer prevention and early detection [149,150].

#### 5.2.2. Major International Consortia

The International Cancer of the Pancreas Screening Consortium (CAPS) and the PRECEDE initiative represent complementary efforts in pancreatic cancer surveillance.

CAPS and PRECEDE are international umbrella consortia that integrate multiple national and institutional high-risk pancreatic cancer registries and surveillance programs. CAPS has primarily focused on consensus-based eligibility criteria and surveillance protocols, while PRECEDE aims to integrate prospective multi-institutional data and biospecimen resources to improve early detection strategies [145,153].

A comparative synthesis of major international and national surveillance programs is provided in Table 2, highlighting substantial heterogeneity in cohort size, eligibility criteria, and outcome reporting. While international consortia have established standardized frameworks, national registries differ markedly in implementation, follow-up intensity, and completeness of survival data, limiting direct cross-program comparisons.

#### 5.2.3. National Registries and Surveillance Programs

Within this landscape, national registries have played a pivotal role in translating genetic risk stratification into structured surveillance pathways. The Italian Registry of Families At Risk of Pancreatic Cancer (IRFARPC) program, supported by the Italian Association for the Study of the Pancreas (AISP), represents a successful example of a coordinated nationwide surveillance initiative. Established in 2015, the registry has enrolled over 1200 high-risk individuals based on familial aggregation and genetic risk factors, demonstrating the feasibility of standardized protocols while contributing to the identification of PDAC at an early, surgically resectable stage [154].

Surveillance protocols in this cohort typically involve annual magnetic resonance cholangiopancreatography (MRCP) and, in selected cases, endoscopic ultrasound (EUS) [155].

Paiella et al. demonstrated that systematic genetic analysis embedded within this program uncovered a substantial proportion of previously unrecognized hereditary risk: 8.8% of high-risk individuals were found to carry PGVs, including mutations in high-penetrance genes [156].

Notably, nearly half of these carriers would not have met conventional testing criteria based solely on family history, underscoring the limitations of restrictive selection approaches and the benefits of structured genetic counseling and cascade testing [156].

Similarly, registries such as the European Registry of Hereditary Pancreatitis and Familial Pancreatic Cancer (EUROPAC) [157], the Spanish Familial Pancreatic Cancer Registry (PANGENFAM) [158], and the Dutch national programs supported by the Netherlands Cancer Registry (NCR) at Leiden University Medical Center and Erasmus MC [159,160] have generated essential data on familial aggregation, variant penetrance, and the diagnostic yield of surveillance.

The PANGENFAM registry has reported that a substantial proportion of enrolled individuals present pancreatic abnormalities during baseline or follow-up imaging, including cystic lesions and other pancreatic changes, and has documented surgically relevant lesions identified during surveillance, alongside a broad spectrum of extra-pancreatic findings, supporting the value of comprehensive imaging-based follow-up in high-risk cohorts [158].

In contrast, evidence from Dutch surveillance programs suggests an evolution toward a more selective, risk-adapted approach for individuals from familial pancreatic cancer kindreds, reflecting refined eligibility criteria and ongoing efforts to optimize benefit–harm balance in surveillance strategies rather than universal screening within all FPC-related high-risk groups [159,160].

A comparative synthesis of major international and national surveillance registries, including cohort size and PDAC outcomes, is provided in Table 2.

**Table 2:** Comparative overview of major international and national pancreatic cancer surveillance registries. Summary of key registry-based surveillance programs, including population size, eligibility criteria, number of pancreatic cancers detected, proportion of early-stage/resectable tumors, and reported clinical outcomes where available. The table highlights heterogeneity across cohorts in surveillance protocols, case ascertainment, and outcome reporting, reflecting differences in study design and risk-enrichment strategies.

Registry/Consortium	Individuals Enrolled	Pancreatic Cancers Detected	Early-Stage Cancers Detected	Evidence of Survival Benefit	Main Contribution
CAPS (CAPS1–5) [146]	1731 HRIs	26 PDACs	57.9% stage I among surveillance-detected cancers	5-year survival 73.3%; median OS 9.8 years vs. 1.5 years outside surveillance	International surveillance framework
CAPS5 [146]	1461 HRIs	10 PDACs	7/9 (77.8%) stage I cancers detected during surveillance	Majority alive at follow-up; supports survival advantage of surveillance	Protocol harmonization and evidence supporting early detection and improved survival.
PANGENFAM [158]	238 HRIs	Not specifically reported	4 individuals underwent surgery for highly suspicious lesions	Long-term surveillance program; survival benefit not yet demonstrated	Registry and biobank
PRECEDE [153]	1759 HRIs	1 PDAC detected at enrollment	Not mature	Not yet available (ongoing prospective study)	Prospective surveillance consortium
IRFARPC [154]	>1200	Not reported	Not reported	Not reported	National surveillance program
EUROPAC (Data derived from aggregated EUROPAC surveillance cohorts reported across multiple studies)	>2000 HRIs enrolled across Europe	Reported across multiple studies	Not consistently reported	Not demonstrated as pooled endpoint	Genotype–phenotype studies

#### 5.2.4. Registry-Based Eligibility Criteria and Classification Frameworks

**Figure 2:**
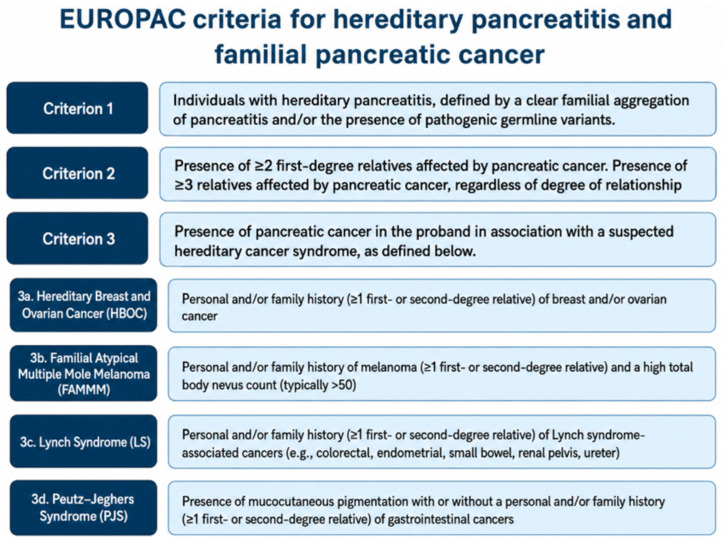
EUROPAC Criteria for the Identification of Hereditary Pancreatitis and Familial Pancreatic Cancer. Overview of the EUROPAC criteria used to identify individuals and families at increased risk for hereditary pancreatitis and familial pancreatic cancer [157].

Different registries and surveillance programs use partially overlapping eligibility criteria to identify individuals at increased risk of PDAC. As an illustrative example, the EUROPAC registry classifies high-risk individuals according to clinical, familial, and genetic criteria (Figure 2).

Specifically, EUROPAC includes: (i) individuals with hereditary pancreatitis, defined by a clear familial aggregation of pancreatitis and/or the presence of PGVs; (ii) individuals with FPC, classically defined as families with PDAC affecting at least two first-degree relatives, or three or more relatives irrespective of the degree of kinship, in the absence of a known hereditary cancer syndrome; and (iii) individuals with hereditary cancer predisposition syndromes associated with an increased risk of PDAC, such as hereditary breast and ovarian cancer (HBOC), familial atypical multiple mole melanoma (FAMMM), Lynch syndrome (LS), and Peutz–Jeghers syndrome (PJS), even when the strict criteria for FPC are not fulfilled. Importantly, fulfilment of any one of these criteria is sufficient for inclusion in the EUROPAC registry. Familial PC represents a clinical definition based on family history, whereas HPC refers to cases in which a PGV is identified; consequently, these terms should not be used interchangeably. This distinction also explains why a proportion of FPC cases do not harbor identifiable PGVs [157] (Table 3).

The risk of individuals within such families increases with the number of affected relatives, with lifetime risk estimates of 18–38% [161].

PGVs are identified in only 10–20% of FPC cases [12], suggesting additional susceptibility genes remain undiscovered. Additional risk factors, such as cigarette smoking, further increase the risk of developing PDAC in individuals with a family history of the disease [161].

Together, these findings suggest a transition toward the implementation of structured, multidisciplinary pathways integrating genetic testing, surveillance, and longitudinal outcome monitoring to evaluate and optimize surveillance effectiveness [162].

### 5.3. Eligibility Criteria for Surveillance

Candidates for pancreatic surveillance, as defined by the 2018 CAPS Consortium, include individuals with PGVs in *STK11*/*LKB1* (PJS) or *CDKN2A* (FAMMM), irrespective of family history; individuals with PGVs in *BRCA2*, *PALB2*, *ATM*, or MMR genes (*MLH1*, *MSH2*, *MSH6*; LS) who have at least one affected first-degree relative; and individuals with at least two affected relatives on the same side of the family, including at least one first-degree relative, regardless of mutation status [9].

Screening is not universally recommended for individuals carrying pathogenic *BRCA1* variants with only one affected FDR. In contrast, AGA guidelines recommend surveillance for carriers of *STK11*, *CDKN2A*, and *PRSS1* mutations irrespective of family history, as well as for carriers of *BRCA1*, *BRCA2*, *PALB2*, *ATM*, or Lynch syndrome-associated genes with at least one affected FDR [110].

Differences between CAPS and AGA recommendations primarily concern individuals with *BRCA1* PGVs, HP, and varying family history patterns. The PDAC risk associated with pathogenic variants in *BRCA1*, *BRCA2*, *PALB2*, or *ATM* in the absence of family history remains incompletely defined. Notably, a study involving 204 *BRCA1/2* PGVs carriers decommissioning comparable rates of pancreatic abnormalities regardless of family history, suggesting a potential role for surveillance independent of familial clustering [163].

In 2013, the CAPS Consortium established that individuals with a lifetime PDAC risk exceeding 5% (or a fivefold increased relative risk) should be considered eligible for surveillance, criteria reaffirmed in the 2020 CAPS update and the AGA expert review [9,110,145].

### 5.4. Age of Surveillance Initiation and Intervals

For individuals from FPC kindreds without identified PGVs, surveillance is recommended to begin at age 50 or 10 years younger than the earliest PDAC diagnosis in the family. For PGVs carriers, surveillance typically begins between ages 45 and 50, or 10 years younger than the youngest affected relative. Earlier initiation is recommended for specific syndromes: (a) *CDKN2A* PGVs carriers: from age 40; (b) PJS: between ages 30 and 40 [9,110].

Despite the challenges posed by severe chronic pancreatitis, individuals with HP should also undergo surveillance beginning at age 40. In cases with low-risk findings assessed by a multidisciplinary team, a 12-month surveillance interval is generally recommended.

### 5.5. Clinical Effectiveness, Goals and Target Lesions of Surveillance Programs

The primary objective of pancreatic surveillance is to reduce mortality from PDAC and to prevent its development through the identification and treatment of precursor lesions. According to AGA, the primary targets are resectable stage I PDAC and high-risk precursor lesions, including IPMNs with high-grade dysplasia and selected enlarged pancreatic intraepithelial neoplasia (PanIN). Published data from surveillance programs demonstrate evidence of downstaging, with the majority of PDACs being diagnosed at stage I or IIB. Detection and management of high-grade dysplasia in PanINs and IPMNs remains a key goal; these high-risk lesions are more frequently identified through pancreatic imaging in individuals with a family history of PDAC and are more likely to represent true precursor lesions compared with those who do not have a similar history [123].

Imaging features of IPMNs can aid in the identification of high-grade dysplasia within these lesions. In contrast, PanINs are predominantly microscopic and cannot be reliably detected with current imaging modalities.

Evidence from prospective cohort studies supports the potential benefit of surveillance in HRIs. In a cohort of 354 HRIs monitored using EUS, magnetic resonance imaging (MRI), and/or computed tomography (CT) over a median follow-up of 5.6 years, 24 individuals (7%) developed neoplasms. Among 14 PDAC cases, 71% were detected through surveillance, with 90% of these being surgically resectable. In contrast, cancers diagnosed outside surveillance programs were symptomatic and unresectable, with significantly lower three-year OS (85% vs. 25%) [164].

Similarly, an MRI-based surveillance study of 79 *CDKN2A* mutation carriers identified PDAC in 9% of participants over a median follow-up of four years, with all detected tumors being resectable at diagnosis, though large prospective studies are still needed to definitively establish survival benefits [165].

### 5.6. Surveillance Modalities and Imaging Strategies

The majority of pancreatic surveillance programs rely on imaging with MRI combined with MRCP and/or EUS, with pancreatic-protocol CT reserved for patients who cannot undergo MRI or EUS due to superior sensitivity for detecting subcentimeter pancreatic cysts and the advantage of avoiding exposure to ionizing radiation [166]. Meta-analyses have not conclusively demonstrated the superiority of one modality over the other for detecting clinically relevant lesions [167,168]. The International CAPS Consortium recommended screening techniques are as follows:

- At baseline: MRI/MRCP plus EUS plus fasting blood glucose and/or glycated hemoglobin.
- During follow-up: alternate MRI/MRCP and EUS; routinely test fasting blood glucose and/or HbA1c to monitor the development of new-onset diabetes.
- As indicated: serum CA 19–9, if concerning features on imaging; EUS-FNA only for solid lesions of ≥5 mm, cystic lesions with worrisome features, or asymptomatic main pancreatic duct (MPD) strictures; CT only for solid lesions, regardless of size, or asymptomatic MPD strictures of unknown etiology.

The consortium recommends a screening interval of every 12 months in patients with no abnormalities, or only non-concerning abnormalities, and every 3 or 6 months in patients with abnormalities that are concerning but not suspicious of malignancy; immediate surgical resection is indicated for suspected malignancy.

According to the AGA, annual imaging is recommended in the absence of suspicious lesions. Low-risk lesions may be followed with EUS at 6–12 months, whereas indeterminate lesions warrant EUS reassessment within 3–6 months and high-risk lesions within 3 months [110].

EUS and MRI have demonstrated good concordance in the assessment of pancreatic lesion size and anatomical location [169] and have been found to be complementary [170], with MRI being particularly sensitive for cystic lesions (such as branch-duct IPMNs) and EUS for solid lesions. Early-stage PDAC may present with subtle imaging features and can be obscured by coexisting conditions like chronic pancreatitis or IPMNs. For these reasons, screening should ideally be conducted in high-volume centers with specialized expertise [169].

Current NCCN clinical guidelines emphasize that imaging must provide a standardized and comprehensive assessment to support accurate staging, documentation of tumor size, arterial/venous involvement, and distant metastases.

Multiphase contrast-enhanced multidetector CT using a pancreas-specific protocol is considered the reference standard for this purpose, while structured reporting is strongly encouraged to facilitate multidisciplinary communication and NCCN resectability criteria application [171,172,173]. Regarding other modalities, transabdominal ultrasonography performance remains suboptimal [174]. CT demonstrates a sensitivity exceeding 90% for solid nodules, but sensitivity decreases to approximately 77% for small tumors (<2 cm), showing inferior performance compared with MRI and EUS in characterizing small cystic lesions [175]. Moreover, radiation exposure limits the use of CT as a primary screening modality, though it remains a viable alternative in patients with MRI contraindications [176]. Table 4 summarizes the key elements of PDAC surveillance in high-risk individuals.

### 5.7. Surveillance Outcomes in Familial and Hereditary Pancreatic Cancer

A meta-analysis of 16 prospective studies involving 1588 HRIs reported resection of target lesions at a rate of 0.5% per patient-year, corresponding to a pooled prevalence of 3.3%. Resection rates varied by syndrome, reaching 12.2% in PJS, 6.3% in HBOC, 5% in FAMMM, 4% in HP, and 3% in FPC [178]. The multicenter Cancer of Pancreas Screening-5 (CAPS5) study demonstrated that 57.9% of PDACs diagnosed through surveillance were stage I, compared with 14.3% diagnosed outside surveillance, showing markedly improved OS (9.8 vs. 1.5 years; adjusted hazard ratio 0.04) and a 5-year survival of 73.3% [146]. Two recent studies from over 20 years of prospective data collection reported encouraging results: Klatte et al. [179] reported that among 347 carriers of a *CDKN2A* PGV, 36 PDACs were detected, with a cumulative incidence of 20.7% by age 70; 83.3% were resectable, with a 5-year survival of 32.4%. These studies highlight the potential benefit of surveillance, although genetic or familial high-risk individuals represent only a small fraction of PDAC cases. In conclusion, current evidence suggests that pancreatic surveillance confers the greatest benefit to individuals carrying high-risk PGVs, whereas its effectiveness in familial PDAC without identified pathogenic variants remains limited. Participation in surveillance programs is also associated with potential harm, such as the inability of imaging to reliably discriminate low-grade from high-grade precursor lesions, and the detection of incidental findings that may lead to unnecessary surgical interventions. Overtreatment represents a significant concern, given that pancreatic resection is associated with substantial perioperative morbidity and long-term exocrine and endocrine dysfunction.

### 5.8. Surveillance Psychological Impact

An individual’s emotional status can significantly influence participation in screening and surveillance programs, while engagement in such programs may, in turn, affect the psychological well-being of participants [180].

Psychosocial impact can be defined as the effect of an intervention on an individual’s social and/or psychological functioning [181].

Concerns have been raised regarding the potential psychological burden of PDAC surveillance in high-risk individuals. While programs have demonstrated the feasibility of detecting early-stage cancers, the potential impact on emotional well-being remains poorly understood. Evidence from surveillance programs for other familial cancers indicates that participation may elicit negative emotional responses, including increased worry and anxiety [182,183], though most participants reported reduced distress and fewer concerns following a negative surveillance examination [184,185].

Available evidence suggests that PDAC surveillance programs are psychologically sustainable. In a prospective study, Paiella et al. reported no clinically significant increase in anxiety or psychological distress among high-risk individuals undergoing surveillance, supporting the emotional feasibility of such programs [180].

Limited evidence suggests that annual PDAC surveillance may even confer positive psychological outcomes [180,186].

In a recent cross-sectional assessment, Anez-Bruzual et al. evaluated the psychosocial impact of surveillance programs, reporting generally low levels of cancer-related distress alongside high perceived benefits and reassurance associated with participation, suggesting that surveillance is overall well tolerated [187].

Identifying whether specific attitudes or beliefs modulate this impact could help to accurately recognize individuals who may benefit from additional psychosocial support, including targeted counseling and education.

## 6. Principles of Pharmacotherapy in Patients with Actionable Genetic Alterations in Hereditary PDAC

Therapeutic management of HPC largely overlaps with that of sporadic disease; however, germline pathogenic variants, particularly in DNA damage repair (DDR) genes such as *BRCA1*, *BRCA2*, *PALB2*, and *ATM*, have important therapeutic implications. Patients harboring germline *BRCA1/2* or *PALB2* mutations demonstrate increased sensitivity to platinum-based chemotherapy due to impaired homologous recombination repair, and such regimens are often preferred in both the advanced and adjuvant settings. Moreover, maintenance therapy with PARPi, notably olaparib, has been shown to significantly prolong progression-free survival in metastatic PDAC patients with *BRCA* PGVs who have not progressed after first-line platinum-based chemotherapy [103,188,189]. Beyond DDR-targeted strategies, ongoing clinical trials are evaluating the role of novel targeted agents and immunotherapeutic approaches in molecularly selected subgroups of HPC, underscoring the importance of universal germline testing to guide personalized treatment strategies and optimize clinical outcomes [188,189,190,191]. Homologous recombination is a DNA double-strand break (DSB) repair mechanism essential for maintaining genomic stability. Key proteins in this pathway include *BRCA1*, *BRCA2*, and *PALB2*, which coordinate the recruitment of *RAD51* to damaged DNA sites and facilitate error-free repair using the sister chromatid as a template. Defects in this pathway lead to homologous recombination deficiency (HRD), resulting in genomic instability and increased vulnerability to DNA-damaging agents.

PARP proteins act as sensors of DNA single-strand breaks (SSBs) and are critical components of the base excision repair (BER) pathway. Inhibition of PARP prevents the repair of SSBs, leading to their conversion into DSBs during DNA replication. In HR-deficient cells, these DSBs cannot be effectively repaired, resulting in cell death through the mechanism of synthetic lethality [192].

However, therapeutic efficacy is highly dependent on the underlying functional status of DNA repair pathways, particularly the presence of biallelic inactivation of homologous recombination repair genes, which represents a key determinant of response to platinum-based chemotherapy and PARP inhibition. In addition, the development of both primary and acquired resistance mechanisms limits long-term efficacy of targeted approaches and remains an important clinical challenge.

### 6.1. Platinum-Based Chemotherapy

The sensitivity of HRD tumors to platinum agents has been well-established in breast and ovarian cancers and is increasingly supported by data in PDAC [193,194]. Platinum-based chemotherapeutic agents induce intra- and interstrand DNA cross-links, particularly between guanine bases, which interfere with DNA replication and transcription and require an intact HR system for repair. Consequently, HRD tumors exhibit increased sensitivity to platinum agents [195]. Importantly, treatment response appears to be strongly influenced by biallelic inactivation of HR genes. Biallelic loss is associated with more profound defects in DNA repair, increased genomic instability, and enhanced sensitivity to platinum-based chemotherapy and PARPi [196]. Preclinical models showed that pancreatic tumors with homozygous *BRCA* mutations are significantly more sensitive to platinum agents than those with heterozygous alterations [197]. In a retrospective analysis involving 71 patients with *BRCA1/2*-associated PDAC, those with unresectable disease who received platinum-based chemotherapy experienced significantly longer OS compared with patients treated with non-platinum regimens (22 vs. 9 months; *p* = 0.039) [198]. A subsequent meta-analysis of six studies confirmed a significant OS benefit for platinum-based therapy in germline *BRCA*-mutated unresectable PDAC (23.7 vs. 12.2 months; mean difference 10.2 months; *p* < 0.001) [199]. Additionally, an analysis of 262 patients undergoing combined germline and somatic testing using the MSK-IMPACT platform demonstrated significantly improved progression-free survival (PFS) with first-line platinum therapy compared to non-platinum regimens in HRD tumors [200]. Whole-genome sequencing analyses have further demonstrated that HRD-associated mutational signatures (notably signature 3) and measures of genomic instability correlate with clinical response to platinum agents in PDAC patients harboring pathogenic variants in *BRCA1/2* or *PALB2* [201]. Based on these data, current NCCN guidelines recommend FOLFIRINOX or modified FOLFIRINOX, or gemcitabine plus cisplatin, as first-line chemotherapy for patients with known *BRCA1/2* or *PALB2* pathogenic variants [112].

### 6.2. PARP Inhibitor Maintenance Therapy

PARP inhibitors have been most extensively studied as maintenance therapy following response to platinum-based chemotherapy. The phase III POLO trial demonstrated that maintenance olaparib significantly prolonged PFS compared with placebo in patients with metastatic PDAC and germline *BRCA1/2* mutations who had not progressed after at least 16 weeks of platinum-based therapy (7.4 vs. 3.8 months; hazard ratio 0.53; *p* = 0.004). The objective response rate (ORR) was also higher in the olaparib group (23% vs. 12%) [188].

Similarly, a phase II study of maintenance rucaparib in platinum-sensitive PDAC patients with germline or somatic pathogenic variants in *BRCA1*, *BRCA2*, or *PALB2* reported a promising ORR of 37% [202].

Response to PARP inhibition is strongly associated with markers of homologous recombination deficiency, including biallelic inactivation of BRCA1/2 or PALB2, mutational signature 3, and genomic instability scores. Conversely, tumors with monoallelic alterations or intact homologous recombination repair mechanisms show limited benefit from PARP inhibitors. Acquired resistance to PARP inhibitors has been increasingly recognized and includes secondary reversion mutations restoring BRCA1/2 function, upregulation of drug efflux pumps, and restoration of homologous recombination competence, all of which contribute to treatment failure and disease progression.

Additional studies have also been conducted to evaluate PARPi as monotherapy, including phase II trials of olaparib and rucaparib in small cohorts of germline *BRCA1/2*-mutated PDAC patients reporting ORRs of approximately 21% [203]. In contrast, veliparib monotherapy showed minimal activity, with a 0% ORR and short median PFS and OS [204]. Moreover, Talazoparib, a next-generation PARPi with strong PARP-trapping activity, demonstrated preliminary activity in a phase I study across multiple *BRCA*-mutated solid tumors [205].

In combination settings, the addition of veliparib to gemcitabine and cisplatin in a randomized phase II trial was associated with a favorable median OS (15.5–16.4 months), further supporting the efficacy of platinum-based chemotherapy in this molecularly defined subgroup [206]. The therapeutic relevance of PARP inhibition in PDAC patients with HRD-related gene alterations beyond *BRCA1/2* (e.g., *ATM*, *CHEK2*, *RAD51*, *FANCA*) remains uncertain. Clinical trials evaluating olaparib monotherapy in this population have demonstrated limited and heterogeneous responses, underscoring the need for improved biomarkers and functional assays [207].

### 6.3. Immune Checkpoint Inhibitors in PDAC

DNA MMR genes found in LS, including *MLH1*, *MSH2*, *MSH6*, and *PMS2*, play a critical role in repairing DNA damage caused by base pair insertions or deletions. Defects in these genes lead to an accumulation of mutations, generating high tumor mutational burden (TMB) [208,209]. High TMB can lead to the expression of tumor-specific neoantigens, which are recognized by the immune system and can be targeted by immunotherapy [210,211]. Immune checkpoint inhibitors (ICIs) have transformed cancer treatment, but their activity in PDAC is limited and mainly restricted to molecularly selected subgroups. The Food and Drug Administration has approved pembrolizumab for the treatment of unresectable or metastatic solid tumors with MSI-H or dMMR [212]. In a multicancer study of 86 patients with dMMR tumors across 12 tumor types, pembrolizumab achieved an ORR of 53.4% and a complete response (CR) rate of 20.9%. In eight PDAC patients included in this cohort, ORR was 62% and CR 25% [213]. Despite encouraging results in biomarker-selected populations, the clinical applicability of immune checkpoint inhibitors in PDAC remains limited, as only approximately 1–2% of patients exhibit MSI-H/dMMR status and response rates are lower than in other gastrointestinal malignancies [214]. Moreover, the immunosuppressive tumor microenvironment of PDAC further restricts therapeutic efficacy in unselected populations. Although homologous recombination deficiency has been proposed as a potential predictive biomarker for immunotherapy response, clinical evidence remains preliminary and restricted to small heterogeneous cohorts, requiring further validation in prospective studies.

Beyond MSI-H/dMMR, HRD may serve as a predictive biomarker for ICI response. HRD tumors harbor defects in DNA repair genes (*BRCA1/2*, *PALB2*, etc.), leading to genomic instability and higher neo-antigen load. In a small series, Terrero et al. reported 12 patients with refractory PDAC or cholangiocarcinoma harboring germline HRD variants treated with ipilimumab plus nivolumab, achieving an ORR of 42%, suggesting HRD may identify patients likely to respond to ICIs [215].

As previously mentioned, combination therapy with PARPi and ICIs also shows potential. In a phase 1b/2 randomized trial, niraparib plus nivolumab or niraparib plus ipilimumab as maintenance therapy after response to platinum-based chemotherapy in PDAC patients with *BRCA* or *PALB2* variants resulted in median PFS of 10.4 months (95% CI, 1.5–19.2) and median OS of 38 months (95% CI not estimable) [216].

### 6.4. Future Perspectives in Hereditary PDAC

Advances in our understanding of risk factors, particularly genetic predispositions, are expected to play a pivotal role in the management of HPC. Increasing knowledge of genetic alterations associated with HPC could help to identify previously unrecognized molecular pathways involved in tumor initiation and progression. This, in turn, may reveal novel actionable targets for pharmacotherapy, enabling more effective precision medicine approaches. Integrating comprehensive genetic profiling into both research and clinical practice will be essential for translating these insights into targeted prevention strategies and individualized therapeutic interventions.

Overall, these data highlight that while molecularly targeted therapies have significantly improved outcomes in selected subgroups of HPC, durable responses remain limited by intrinsic tumor heterogeneity, emergence of resistance mechanisms, and the restricted proportion of patients eligible for immunotherapy or targeted approaches. This underscores the need for improved predictive biomarkers and functional assays to refine patient selection and optimize therapeutic strategies.

## 7. Future Perspectives

PDAC remains one of the most aggressive solid malignancies, with late-stage diagnosis and poor prognosis. Traditional biomarkers such as CA 19-9 and CEA offer limited sensitivity and specificity, particularly in early-stage disease [217]. Emerging technologies are reshaping the biomarker landscape through liquid biopsy platforms, including circulating tumor DNA, circulating tumor cells, and exosomes; and high-throughput genomic, proteomic, metabolomic, and epigenetic profiling. Future efforts will increasingly rely on integrated, multi-analyte strategies that combine these molecular layers into unified diagnostic frameworks. Advances in single-cell technologies and spatial multi-omics will deepen understanding of tumor heterogeneity and microenvironmental interactions, revealing new biomarker candidates and therapeutic vulnerabilities. Artificial intelligence and machine-learning models will play a pivotal role in integrating these high-dimensional datasets, enhancing predictive accuracy and supporting clinical decision-making. Standardization of pre-analytical workflows, assay harmonization, and large-scale prospective validation will be essential to translate these innovations into routine clinical practice [217]. Ultimately, the synergy of technological innovation, rigorous validation, and structured translational frameworks has the potential to transform PDAC management from reactive treatment toward a proactive, precision-driven paradigm. By integrating high-dimensional molecular data, advanced imaging, and clinical insights within coordinated high-risk cohorts and registries, it may become possible to identify the earliest signs of disease, refine individualized risk stratification, and guide personalized interventions before overt malignancy develops. This convergence of innovation and translational rigor not only promises earlier detection and improved patient outcomes but also opens the door to a truly anticipatory approach to PDAC, where prevention, surveillance, and therapy are dynamically tailored to each patient’s unique molecular and clinical profile.

## 8. Conclusions

Hereditary and familial PC accounts for a significant minority of PDAC cases, with up to 10% of patients carrying PGVs or a strong family history. Early identification of HRIs through genetic testing and structured surveillance programs is essential to improving outcomes. Collectively, registries and consortia have enabled a paradigm shift from a purely descriptive definition of familial risk toward a more sophisticated, genomically informed model. Their contributions are now central to the development of early detection strategies, the identification of actionable precursor lesions, and the implementation of truly personalized prevention pathways for individuals at elevated risk of PDAC. Current screening strategies, primarily using MRI and/or EUS in specialized centers, aim to detect PDAC at a stage when curative interventions are possible and to identify high-grade precursor lesions such as PanIN and IPMN. However, the detection of microscopic lesions remains a major challenge, and surveillance carries potential risks including false positives, overtreatment, and psychological burden. Screening recommendations are increasingly tailored to specific genetic backgrounds: patients with *LKB1/STK11* or *CDKN2A* PGVs should undergo screening regardless of family history, whereas carriers of PGVs in *BRCA1/2*, *PALB2*, *ATM*, *MLH1*, *MSH2*, or *MSH6*, as well as those with FPC, should be screened based on the presence of affected first-degree relatives, with age of initiation adjusted according to syndrome-specific risk and the youngest affected family member. Annual MRI or EUS remain the cornerstone of imaging-based surveillance. Future research should focus on refining risk stratification, discovering reliable biomarkers, and developing advanced imaging techniques to enable earlier detection of both precursor lesions and invasive PDAC. Optimizing these approaches holds the potential to improve prognosis and guide personalized management in individuals with HPC, ultimately translating genetic and molecular insights into tangible clinical benefit.

## Figures and Tables

**Table 1:** Clinical indications and genetic testing criteria for hereditary cancer syndromes (according to NCCN guidelines).

Syndrome	Main Gene(s)	Clinical Indications for Testing
Lynch Syndrome (HNPCC) [105]	*MLH1*, *MSH2*, *MSH6*, *PMS2*, *EPCAM*	Early-onset Lynch-associated cancers; suggestive family history; abnormal MSI/IHC testing.
Hereditary Breast and Ovarian Cancer (HBOC) [42]	*BRCA1*, *BRCA2*	Breast, ovarian, pancreatic or aggressive prostate cancer; family clustering; Ashkenazi ancestry.
FAMM Syndrome [61]	*CDKN2A*, *CDK4*	Multiple atypical nevi; familial melanoma; melanoma-pancreatic cancer aggregation.
Peutz–Jeghers Syndrome (PJS) [106]	*STK11*	Hamartomatous polyps; mucocutaneous pigmentation; family history of PJS.
Familial adenomatous polyposis (FAP) [107]	*APC*	Multiple adenomatous polyps; APC-positive family history; characteristic extracolonic manifestations.

**Table 3:** Overview of population-based registries, high-risk surveillance programs, and international consortia in familial pancreatic cancer. The table provides a comprehensive overview of target populations, types of registries and programs, and key characteristics, including data collected, surveillance protocols, and the scope of each initiative on a national and international scale.

Major Familial Pancreatic Cancer Registries and High-Risk Surveillance Programs					
Registry/ Program	Country/ Region	Type	High-Risk Population	Main Contribution	Key Outcomes
IRFARPC [154,155]	Italy	National Registry	- FPC: ≥2 or ≥3 relatives with PC, even without known genetic variants. - Predisposing genetic variants: CDKN2A, STK11, PRSS1, without family history, etc.; BRCA1/2, PALB2, LS, AT, TP53 with family history.	- National surveillance program	>1200 enrolled; structured surveillance; outcome data emerging
EUROPAC [157]	Europe	Multicenter Registry	- FPC: individuals with HPC/FPC (≥2 first-degree relatives) - Hereditary syndromes: include classic genetic syndromes (e.g., PJS, HBOC, FAMMM, LS), hereditary pancreatitis, and other hereditary pancreatic diseases.	- Risk stratification and genotype–phenotype studies	Long-term familial risk characterization; surveillance outcomes reported in selected cohorts
PANGENFAM [158]	Spain	National Registry	- FPC: strongly centered on FPC. Typically, ≥2 affected relatives (often with first-degree relatives being affected). - Hereditary syndromes: Include inherited syndromes associated with pancreatic ductal adenocarcinoma risk.	- Early detection screening; research, biobanking	Genotype–phenotype characterization and biobanking
NCR [160]	Netherlands	Population-based cancer registry	All incident cancer cases in the Netherlands	- National epidemiological database with high completeness and data quality; supports outcome and population-level analyses	Increased detection of stage I/resectable PC and improved survival compared with historical controls
NATIONAL HIGH-RISK PC SURVEILLANCE PROGRAMS (LEIDEN UNIVERSITY MEDICAL CENTER; ERASMUS MC) [160]	Netherlands	High-risk surveillance program	- FPC: Includes subjects with FPC. Typically, ≥2 affected relatives at least one first-degree relative. - Genetic variants - Hereditary cancer syndromes associated with pancreatic cancer risk	- Structured imaging-based surveillance and longitudinal follow-up of genetically predisposed individuals	Increased detection of stage I/resectable PC and improved survival compared with historical controls
CAPS [145]	International	Surveillance Consortium	- FPC: families with ≥2 affected first-degree relatives or strong family history: ≥3 affected relatives (with at least 1 first-degree) - PGV carriers + family history - Hereditary Syndromes	- Consensus guidelines and protocol harmonization	Early-stage PC detection and survival benefit demonstrated in high-risk individuals
PRECEDE [153]	International	Surveillance Consortium	- FPC: individuals with FPC. Typically, ≥2 affected relatives. Emphasis on first-degree relatives. - PGV carriers + family history - Genetic variants: *BRCA1/2*, *CDKN2A*, *STK11*, *PALB2*, and other known predisposition genes. Enrollment may be included even without family history. - Hereditary Syndromes	- Prospective surveillance and biospecimen collection	Ongoing prospective cohort; outcome data not yet mature

**Table 4:** Key components of pancreatic cancer surveillance in high-risk individuals. The table summarizes target populations, age at surveillance initiation, preferred imaging modalities, and follow-up intervals based on current international recommendations, including those from the CAPS Consortium and the American Gastroenterological Association. * Surveillance is generally recommended when at least one first-degree relative is affected. Notes: PanIN is often undetectable; IPMN may show high-grade dysplasia [9,110,123,163,166,177].

Pancreatic Cancer Surveillance in High-Risk Individuals	
Category	Key Points
Target population (Who)	- High-risk individuals defined by germline pathogenic variants (e.g., CDKN2A, STK11/PJS, PRSS1, BRCA1/2, PALB2, ATM, Lynch syndrome *). - Familial pancreatic cancer (≥2 affected relatives, including ≥1 first-degree relative).
Age initiation (When)	- CDKN2A, hereditary pancreatitis: 40 years. - Peutz–Jeghers syndrome: 30–40 years. - Other PVs or familial PC: 45–50 years or 10 years earlier than the youngest affected relative.
Preferred modalities (How)	- Primary imaging: MRI/MRCP and/or EUS (preferred for cystic and solid lesions, respectively). - CT: reserved for MRI/EUS contraindications or solid lesion characterization. - Laboratory markers: CA 19-9 if concerning imaging features, fasting glucose/HbA1c routinely. - EUS-FNA if: Solid lesions ≥5 mm; Cystic lesions with worrisome features; MPD strictures (±mass).
Follow-up (Intervals)	- No concerning lesions: Annual imaging surveillance. - Low-risk lesions: EUS at 6–12 months, then annual surveillance. - Indeterminate lesions: EUS reassessment within 3–6 months. - High-risk lesions: EUS within 3 months. Surgical evaluation if malignancy suspected.

## Data Availability

No new data were created or analyzed in this study. Data sharing is not applicable to this article.

## Abbreviations

The following abbreviations are used in this manuscript:

ACG	American College of Gastroenterology
AGA	American Gastroenterological Association
AISP	Italian Association for the Study of the Pancreas
ASCO	American Society of Clinical Oncology
AT	Ataxia–Telangiectasia
BD-IPMN	Branch-Duct IPMN
BER	Base Excision Repair
CAPS	International Cancer of the Pancreas Screening
CDKN2A	Cyclin-Dependent Kinase Inhibitor 2A
CFTR	Cystic Fibrosis Transmembrane Conductance Regulator
CI	Confidence Interval
CR	Complete Response
CT	Computed tomography
DDR	DNA Damage Repair
dMMR	Mismatch Repair Protein Deficiency
DPCA	Dutch Pancreatic Cancer Audit
DSB	Double-Strand Break
EUROPAC	European Registry of Hereditary Pancreatitis and Pancreatic Cancer
EUS	Endoscopic Ultrasonography
FAMM	Familial Atypical Multiple Mole Melanoma
FAP	Familial Adenomatous Polyposis
FNA	Fine-Needle Aspiration
FNB	Fine-Needle Biopsy
FPC	Familial Pancreatic Cancer
GenoMEL	Melanoma Genetics Consortium
HBOC	Hereditary Breast and Ovarian Cancer
HP	Hereditary Pancreatitis
HPC	Hereditary Pancreatic Cancer
HR	Homologous Recombination
HRD	Homologous Recombination Deficiency
HRHR	Homologous Recombination
HRI	High-Risk Individual
ICI	Immune Checkpoint Inhibitor
IPMN	Intraductal Papillary Mucinous Neoplasm
IRFARPC	Italian Registry of Families At Risk of Pancreatic Cancer
LAPCR	Locally Advanced Pancreatic Cancer Registry
LFS	Li–Fraumeni Syndrome
LS	Lynch Syndrome
MD-IPMN	Main Duct-IPMN
MLH1	MutL Homolog 1
MMR	Mismatch Repair
MRCP	Magnetic Resonance Cholangiopancreatography
MRI	Magnetic Resonance Imaging
MSH2	MutS Homolog 2
MSH6	MutS Homolog 6
MSI	Microsatellite Instability
MSI-H	MSI-High
MSI-I	MSI-Indeterminate
MSS	Microsatellite Stable
NCCN	National Comprehensive Cancer Network
NCR	Netherlands Cancer Registry
OGV	Pathogenic Germline Variant
ORR	Objective Response Rate
OS	Overall Survival
PANGENFAM	Spanish Familial Pancreatic Cancer Registry
PanIN	Pancreatic Intraepithelial Neoplasia
PARPi	Poly-(ADP-ribose) Polymerase inhibitor
PC	Pancreatic Cancer
PFS	Progression-Free Survival
PGPV	Presumed Germline Pathogenic Variant
PGV	Pathogenic Germline Variants
PJS	Peutz–Jeghers Syndrome
PMS-2	Post-Meiotic Segregation increased 2
PRECEDE	Pancreatic Cancer Early Detection Consortium
PRSS1	Protease Serine 1
RER+	Replication Error—positive
RR	Relative Risk
SEER	Surveillance, Epidemiology, and End Results
SPINK1	Serine Protease Inhibitor Kazal type 1
SSB	Single-Strand Breaks
STK11	Serine/Threonine Kinase 11
TMN	Tumor Mutational Burden
